# IL-21 Modulates Release of Proinflammatory Cytokines in LPS-Stimulated Macrophages through Distinct Signaling Pathways

**DOI:** 10.1155/2013/548073

**Published:** 2013-12-26

**Authors:** Su-nan Li, Wei Wang, Shou-peng Fu, Jian-fa Wang, Hong-mei Liu, Shan-shan Xie, Bing-run Liu, Yang Li, Qing-kang Lv, Zhi-qiang Li, Wen-jing Xue, Bing-xu Huang, Wei Chen, Ju-xiong Liu

**Affiliations:** ^1^College of Veterinary Medicine, Jilin University, Changchun 130062, China; ^2^College of Animal Science and Veterinary Medicine, Heilongjiang Bayi Agricultural University, Daqing 163319, China

## Abstract

The aim of this study was to investigate the anti-inflammatory effect of IL-21 on LPS-induced mouse peritoneal macrophages. The results showed that IL-21 significantly inhibited LPS-induced mRNA expression of IL-1**β**, TNF-**α**, and IL-6 in macrophages, but not of IFN-**γ**, IL-10, CCL5, or CXCL2. ELISA analysis showed that IL-21 also suppressed LPS-induced production of TNF-**α** and IL-6 in culture supernatants. Western blot analysis showed that IL-21 clearly inhibited ERK and I**κ**B**α** phosphorylation and NF-**κ**B translocation in LPS-stimulated macrophages, but it increased STAT3 phosphorylation. Flow cytometric and Western blot analysis showed that IL-21 decreased M1 macrophages surface markers expression of CD86, iNOS, and TLR4 in LPS-stimulated cells. All results suggested that IL-21 decreases IL-6 and TNF-**α** production via inhibiting the phosphorylation of ERK and translocation of NF-**κ**B and promotes a shift from the M1 to M2 macrophage phenotype by decreasing the expression of CD86, iNOS, and TLR4 and by increasing STAT3 phosphorylation in LPS-stimulated cells.

## 1. Introduction

Interleukin-21 (IL-21) is produced by activated CD4^+^ T-cells, natural killer T cells (NKT cells), and follicular T helper cells. The IL-21 receptor was discovered in 2000 as an orphan receptor, first denoted as NILR for novel interleukin receptor and now as IL-21R [1, 2]. IL-21 receptor expression has been detected on CD4^+^ T cells, CD8^+^ T cells, B cells, NK cells, macrophages, and dendritic cells (DCs) [1–6], suggesting that IL-21 has a broad range of functions. In addition, the IL-21 receptor is a member of a family of receptors that share the *γ* chain (*γ*c). Analogous to the other *γ*c family cytokines, IL-21 activates both Jak1 and Jak3 [1, 7, 8], and weakly activates Stat5 proteins [9]. Stat3 appears to be the most important STAT protein for IL-21 signaling. In addition, the phosphoinositol 3-kinase/Akt (PI3K/Akt) and Ras/MAP kinase (MAPK) pathways also contribute to IL-21 signaling [10]. IL-21 also clearly has an important effect on B cells, T cells, and NK T cells. For example, IL-21 can augment anti-CD40-induced human B-cell proliferation, but it inhibits proliferation to anti-IgM and IL-4 [2] and can increase the proliferation of NK T cells in response to *in vitro* stimulation with anti-CD3, but only when combined with either IL-2 or IL-15 [11].

Macrophages are important innate immune cells that are strategically located throughout the body tissues, where they ingest and process foreign materials, dead cells, and debris and recruit additional macrophages in response to inflammatory signals. They are highly heterogeneous cells that can rapidly change their function in response to local microenvironmental signals (including infection and injury). Differentially activated macrophages display distinct functional phenotypes [12–14]. Macrophages stimulated with toll-like receptor (TLR) ligands, such as lipopolysaccharide (LPS) and/or IFN-*γ*, are termed as classical activation macrophages (M1 macrophages) [12–14], whereas activation by Th2 cytokines such as IL-4 and IL-13 generates alternatively activated macrophages (M2 macrophages) [12–15]. M1 macrophages mediate defense of the host from a variety of bacteria, protozoa, and viruses, and have roles in antitumor immunity. M2 macrophages have anti-inflammatory functions and regulate wound healing. Most importantly, M1 and M2 phenotypes might not be stably differentiated subsets in the same way as, for example, Th1 and Th2 cells. The polarized activation of macrophages has been extensively studied at the transcriptional level [12]. NF-*κ*B, AP-1, PU.1, CCAAT/enhancer-binding protein *α* (C/EBP-*α*), and IFN-regulatory factor 5 (IRF5) have been shown to mediate M1 activation by TLR ligands.

LPS is a major component of the outer membrane of Gram-negative bacteria and stimulates the host immune response upon interaction with the pattern-recognition receptor TLR expressed on host cells. LPS activates NF-*κ*B and the MAPK family, which are classified into at least three components: extracellular signal-regulated kinases (ERKs), c-Jun N-terminal kinase (JNK), and p38 MAPK, which have been implicated in the release of immune-related cytotoxic factors such as inducible NO synthase (iNOS), cyclooxygenase (COX)-2, and proinflammatory cytokines such as TNF-*α*, IL-1*β*, and IL-6 [12–14]. IL-21 has been reported to have an important role in immune response. However, there is little information available on whether IL-21 is able to exert anti-inflammatory effects on LPS-induced macrophages.

The present study was designed to investigate the anti-inflammatory effects and mechanisms of IL-21 in the LPS-induced inflammatory responses in mouse peritoneal macrophages. We studied the mRNA expression and protein secretion of cytokines and chemokines and the activity of two signal pathways, MAPKs and NF-*κ*B, which are activated by TLR4 and responsible for the regulation of the intracellular secretion of proinflammatory cytokine. In addition, we also studied the expression of M1 macrophage surface markers such as CD86, iNOS, and TLR4 and the phosphorylation of STAT3 to confirm whether IL-21 can affect population of M1 macrophages in LPS-stimulated cells.

## 2. Materials and Methods

### 2.1. Animals

Female Balb/c mice (18–22 g, 6–8 weeks) were purchased from the Center of Experimental Animals of Bethune Medical College of Jilin University (Jilin, China). The mice were housed under constant temperature and humidity with 12-hour light-dark cycle and were given free access to food and water. All procedures were approved by the Protection of Vertebrate Animals used for Experimental and other Scientific Purposes.

### 2.2. Reagents

Recombinant murine IL-21 was purchased from PEPROTECH, and Lipopolysaccharide (LPS, *Escherichia coli* 055:B5) and thioglycollate broth were purchased from Sigma Chemical Co. (St. Louis, MO, USA). RPMI 1640, Fetal Bovine Serum (FBS), Trizol Reagent, and the Alexa Fluor 488 Annexin V/Dead Cell Apoptosis Kit were obtained from Invitrogen (Carlsbad, USA). Mouse TNF-*α*, IL-1*β*, and IL-6 enzyme-linked immunosorbent assay (ELISA) kits and Alexa Fluor 488 anti-mouse CD86, PE-Rat IgG2b, and PE-anti-mouse/human CD11b antibodies were purchased from Biolegend (CA, USA). Antibodies to TLR4, phospho-JNK (Thr-183/Tyr-185), JNK, phospho-p38 MAPK (Thr-180/Tyr-182), p38 MAPK, phospho-STAT3, STAT3, phospho-p44/42 ERK1/2, p44/42 ERK1/2, phospho-I*κ*B-*α* (Ser 32), and I*κ*B-*α* were purchased from Cell Signaling Technology, Inc. (Beverly, MA). Antibodies to NF-*κ*B p65, PCNA, Goat anti-rabbit IgG-HRP, Rabbit anti-goat IgG-HRP, iNOS, and Actin(1-19) were purchased from Santa Cruz Biotechnology (Santa Cruz, CA).

### 2.3. Cell Culture

The mice were intraperitoneally injected with 4 mL of 3% thioglycollate broth. Four days later, the mice were sacrificed. Peritoneal macrophages were isolated by lavage of the peritoneal cavity with RPMI 1640 supplemented with 10% fetal bovine serum and collected by centrifugation. Cells were then cultured in RPMI 1640 at a density of 1 × 10^7^ cells/mL in 60 mm culture dishes or 1 × 10^6^ in 24-well tissue culture plates supplemented with 10% fetal bovine serum, 1% penicillin and streptomycin in a 37°C, and 5% CO_2_ incubator. Two hours later, nonadherent cells were discarded. The remaining adherent cells were cultured overnight until they were used for the experiments. In all experiments, macrophages were incubated with IL-21 (100 ng/mL) and/or LPS (100 ng/mL) for different time points.

### 2.4. Real Time RT-PCR

Macrophages (1 × 10^6^/well) were incubated with IL-21 and/or LPS for 3 h, 6 h, and 24 h. Total RNA was isolated from cells using Trizol Reagent according to the manufacturer's protocol. The mRNA levels of various genes were quantified using the SYBR Green QuantiTect RT-PCR Kit (Roche, South San Francisco, CA, USA). GAPDH was used as an endogenous reference. Data were analyzed using the relative standard curve method according to the manufacturer's protocol. A mean value of each gene after GAPDH normalization at the time point showing the highest expression was used as a calibrator to determine the relative levels of IL-21R, *γ*c, TNF-*α*, IL-10, IL-1*β*, IL-6, IFN-*γ*, iNOS, CXCL2, TLR4, and CCL5 at different time points. In addition, PCR products were resolved on a 1.5% agarose gel and stained with ethidium bromide. The primer sequences for the tested genes were the following:


GeneSequenceGAPDH-Forward Primer5′-ACCACAGTCCATGCCATCAC-3′GAPDH-Reverse Primer5′-TCCACCACCCTGTTGCTGTA-3′IL-21R-Forward Primer5′-ACAACAACATCAGCCTTACA-3′IL-21R-Reverse Primer5′-AACAGACCAAATCCCAACA-3′
*γ*c-Forward Primer5′-GTCGACAGAGCAAGCACCATGTTGAAACTA-3′
*γ*c-Reverse Primer5′-GGATCCT GGGATCACAAGATTCTGTAGGTT-3′IL-1*β*-Forward Primer5′-CTCGGCCAAGACAGGTCGCTC-3′IL-1*β*-Reverse Primer5′-CCCCCACACGTTGACAGCTAGG-3′iNOS-Forward Primer5′-GAACTGTAGCACAGCACAGGAAAT-3′iNOS-Reverse Primer5′-CGTACCGGATGAGCTGTGAAT-3′IFN-*γ*-Forward Primer5′-TGAGACAATGAACGCTAC-3′IFN-*γ*-Reverse Primer5′-TTCCACATCTATGCCACT-3′CXCL2-Forward Primer5′-CACCAACCACCAGGCTAC-3′CXCL2-Reverse Primer5′-CTTCAGGGTCAAGGCAAA-3′CCL5-Forward Primer5′-ACCACTCCCTGCTGCTTT-3′CCL5-Reverse Primer5′-ACACTTGGCGGTTCCTTC-3′TNF-*α*-Forward Primer5′-CTGTGAAGGGAATGGGTGTT-3′TNF-*α*-Reverse Primer5′-CAGGGAAGAATCTGGAAAGGTC-3′IL-6-Forward Primer5′-TTCTTGGGACTGATGCTG-3′IL-6-Reverse Primer5′-CTGGCTTTGTCTTTCTTGTT-3′IL-10-Forward Primer5′-GGAGGGGTTCTTCCTTGGGA-3′IL-10-Reverse Primer5′-GTTTTCAGGGATGAAGCGGC-3′TLR4-Forward Primer5′-GAAACGGCAACTTGGACCTG-3′TLR4-Reverse Primer5′-ATGTGTTCCATGGGCTCTCG-3′


### 2.5. ELISA Analysis

Macrophages (1 × 10^6^/well) were incubated with IL-21 and/or LPS for 6 h, 12 h, and 24 h. Cell-free supernatants were subsequently employed for TNF-*α*, IL-10, IL-1*β*, and IL-6 assays using mouse enzyme-linked immunosorbent assay (ELISA) kits according to the manufacturer's instructions.

### 2.6. Western Blot Analysis

Macrophages (1 × 10^7^/60-mm dish) were incubated with IL-21 and/or LPS for 5 min, 20 min, and 60 min. The cells were collected and washed twice with cold PBS. The suspension was mixed with buffer A (10 mM HEPES, pH 7.5, 10 mM KCl, 0.1 mM EGTA, 0.1 mM EDTA, 1 mM DTT, 0.5 mM PMSF, 5 *μ*g/mL aprotinin, 5 *μ*g/mL pepstatin, and 10 *μ*g/mL leupeptin) and lysed by three freeze-thaw cycles. The cytosolic fraction was obtained by centrifugation at 12,000 ×g for 20 min at 4°C. The pellets were re-suspended in buffer B (20 mM HEPES, pH 7.5, 0.4 M NaCl, 1 mM EGTA, 1 mM EDTA, 1 mM DTT, 1 mM PMSF, 5 *μ*g/mL aprotinin, 5 *μ*g/mL pepstatin, and 10 *μ*g/mL leupeptin) on ice for 40 min and centrifuged at 14,000 ×g for 20 min at 4°C. The resulting supernatant was used as the soluble nuclear fraction. In some experiments, the cell suspension was lysed with lysis buffer according to the manufacturer's instructions (Beyotime, Jiangsu, China). Protein content was determined with the BCA protein assay reagent. Proteins (20–30 *μ*g) were subjected to electrophoresis in 10% SDS-polyacrylamide gels and transferred onto a polyvinylidene fluoride (PVDF) membrane with glycine transfer buffer (192 mM glycine, 25 mM Tris-HCl (pH 8.8), 20% methanol (v/v)). After blocking the nonspecific site with blocking solution (5% (wt/vol) nonfat dry milk), the membrane was incubated overnight with a specific primary antibody at 4°C. The membrane was then incubated for an additional 60 min with a peroxidase-conjugated secondary antibody at room temperature. The immunoactive proteins were detected using an enhanced chemiluminescence (ECL) Western blotting detection kit.

### 2.7. Flow Cytometry

Cells were seeded into 6-well plates at a density of 1 × 10^6^ cells per well and were treated with various concentrations of IL-21 (100 ng/mL). After 24 h, the media were aspirated and cells were washed with PBS twice. Then the cells were stained with FITC-conjugated Annexin V and propidium iodide (PI) using Annexin V-FITC Apoptosis Detection kit according to the manufacturer's recommendation (Calbiochem). Flow cytometry (BD Biosciences, USA) was used to determine the percentage of apoptotic cells.

In addition, M1 macrophage surface markers of CD11b and CD86 were measured by flow cytometry. The cells were harvested, washed, and incubated with Alexa Fluor 488 anti-mouse CD86 antibody and PE anti-mouse/human CD11b antibody for 30 min after treatment with IL-21 and/or LPS for 24 h. Flow cytometry (BD Biosciences, USA) was used to determine the population of CD11b^+^CD86^+^ cells.

### 2.8. Statistical Analysis

Dates were analyzed using GraphPad Prism 5 (GraphPad InStat Software, San Diego, CA, USA). Comparison among groups was made with ANOVA followed by Dunnett's test. Data are presented as mean ± SD. *P*-values of 0.05 or less were considered statistically significant difference (^#^ *P* < 0.001 significantly different from the NT group; *P < 0.05, **P < 0.01 compared with the LPS group).

## 3. Results

### 3.1. IL-21 Induces the mRNA Expression of IL-21R in Mouse Macrophages

**Figure 1:**
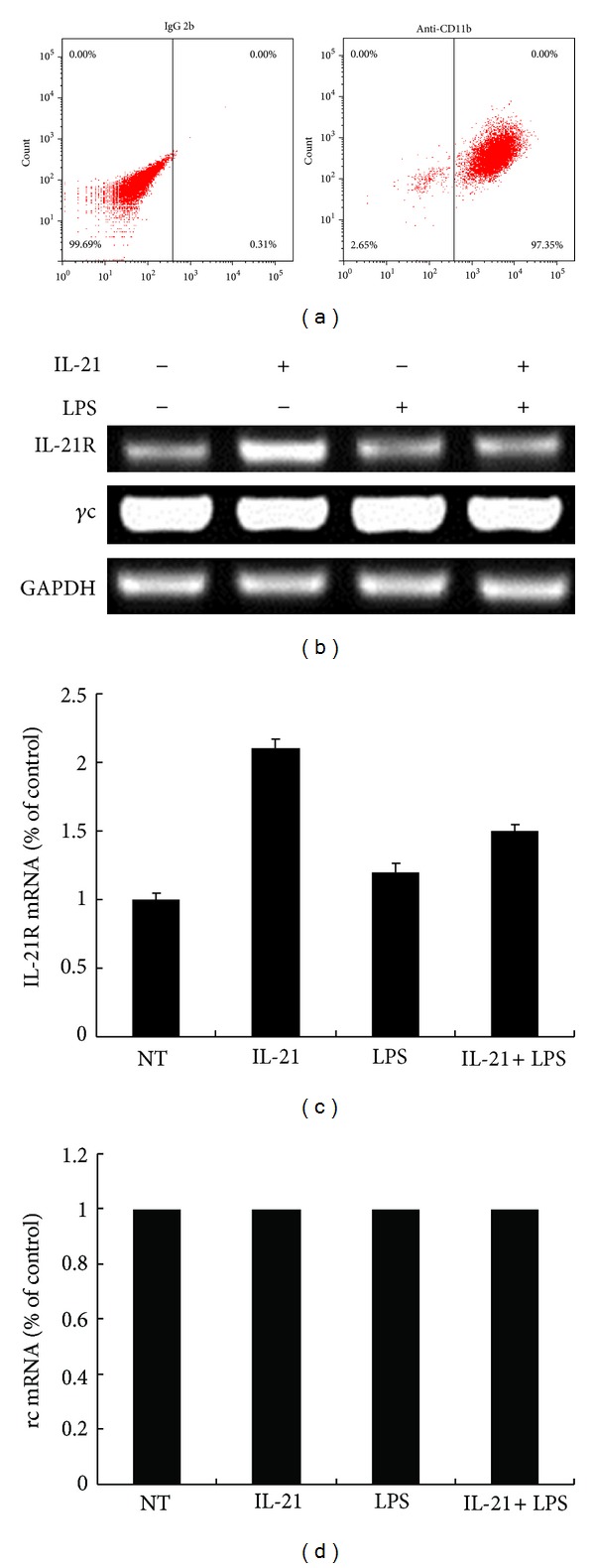
Effects of IL-21 on LPS-induced IL-21R and *γ*c mRNA expression in mouse peritoneal macrophages. Mouse peritoneal macrophages were incubated with IL-21 (100 ng/mL) in the presence or absence of LPS (100 ng/mL) for 6 h. Macrophages were stained with IgG2b and CD11b antibodies (a). Total RNA was isolated and analyzed by real time RT-PCR for IL-21R (c) and *γ*c (d). IL-21R and *γ*c expression was normalized to endogenous control GAPDH. Results are expressed as mean ± SD for each group from three independent experiments.

In our study, more than 97% of the adherent cell population was macrophages as determined by the specific surface marker CD11b by flow cytometry (Figure 1(a)). The functional receptor for IL-21 is IL-21R + *γ*c [6, 7]. To study the biological effects of IL-21, we first evaluated the expression of IL-21R and *γ*c in macrophages by RT-PCR. It is surprising that IL-21R expression was increased, induced by IL-21 alone, compared to the control group and the LPS group (Figures 1(b) and 1(c)). However, *γ*c expression was high in macrophages, and no significant difference was detected after treatment with IL-21 and/or LPS in 6 h (Figures 1(b) and 1(d)).

### 3.2. IL-21 Inhibited LPS-Induced Cytokine and Chemokine mRNA Expression in Macrophages

**Figure 2:**
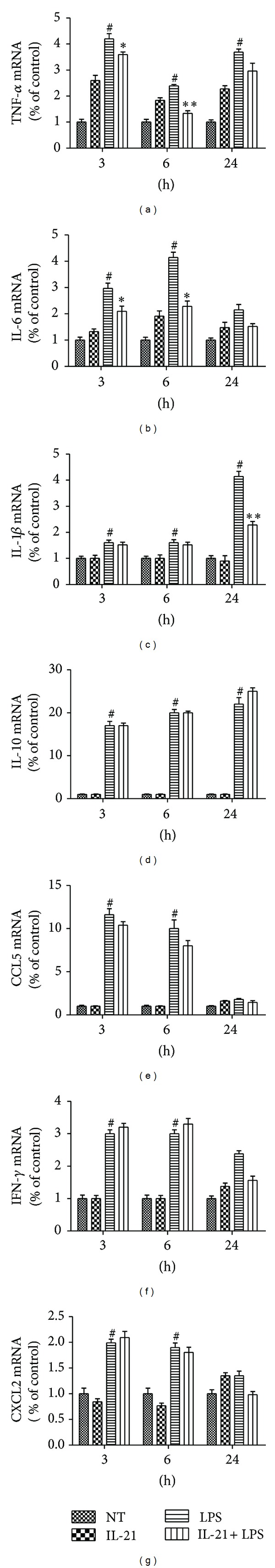
Effects of IL-21 on LPS-induced cytokine and chemokine mRNA expression in mouse peritoneal macrophages. Macrophages were incubated with IL-21 (100 ng/mL) in the presence or absence of LPS (100 ng/mL) for 3 h, 6 h, and 24 h. Total RNA was isolated and analyzed by real time RT- PCR for TNF-*α* (a), IL-6 (b), IL-1*β* (c), IL-10 (d), CCL5 (e), IFN-*γ* (f), and CXCL2 (g) mRNA expression. All of these expressions were normalized to endogenous control GAPDH. Results are expressed as mean ± SD for each group from three independent experiments. ^#^ *P* < 0.001 significantly different from the NT group; *P < 0.05, **P < 0.01 compared with the LPS group.

A conventional approach of studying macrophage activation *in vitro* is to stimulate cells with LPS and then measure the effector cytokine and chemokine production and changes in gene expression. Thus, we investigated whether IL-21 treatment inhibited the gene expression of cytokines and chemokines. The results showed that treatment of macrophages with LPS alone resulted in a significant increase in cytokine expression compared to the control group (*P* < 0.01). However, IL-21 significantly inhibited LPS-induced expression of TNF-*α*, IL-6, and IL-1*β* compared to the LPS group (*P* < 0.05 or *P* < 0.01, Figures 2(a)–2(c)), but the expression of IL-10, CCL5, IFN-*γ*, and CXCL2, which were secreted by activated macrophages, was not suppressed by IL-21 (Figures 2(d)–2(g)).

### 3.3. IL-21 Decreased LPS-Induced Production of TNF-*α* and IL-6 in Cell Culture Supernatants

**Figure 3:**
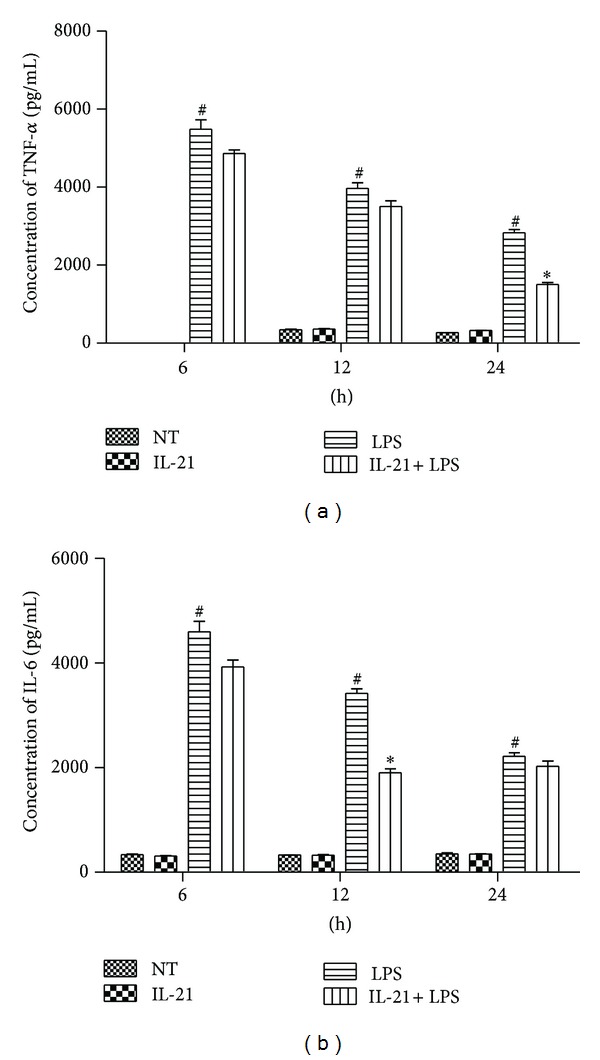
Effects of IL-21 on LPS-induced TNF-*α* and IL-6 secretion in mouse peritoneal macrophage culture supernatants Macrophages were incubated with IL-21 (100 ng/mL) in the presence or absence of LPS (100 ng/mL) for 6 h, 12 h and 24 h. The concentration of TNF-*α* (a) and IL-6 (b) were measured by ELISA. Values are presented as mean ± SD of three independent experiments. ^#^ *P* < 0.001 significantly different from the NT group; *P < 0.05, **P < 0.01 compared with the LPS group.

Macrophage activation was accompanied by cytokine secretion. Here, we demonstrate that IL-21 treatment inhibited the LPS-induced mRNA expression of TNF-*α*, IL-6, and IL-1*β*. Next, their concentrations in the culture supernatants of macrophages were measured by sandwich ELISA. Treatment of macrophages with LPS alone resulted in a remarkable increase in cytokine production compared to the NT group (*P* < 0.001). However, IL-21 treatment significantly inhibited the LPS-induced production of TNF-*α* and IL-6 compared to the LPS group (*P* < 0.05, Figures 3(a) and 3(b)). The secretion of IL-1*β* could not be detected in the cell culture supernatants.

### 3.4. IL-21 Clearly Inhibited the Phosphorylation of ERK in LPS-Induced Macrophages

**Figure 4:**
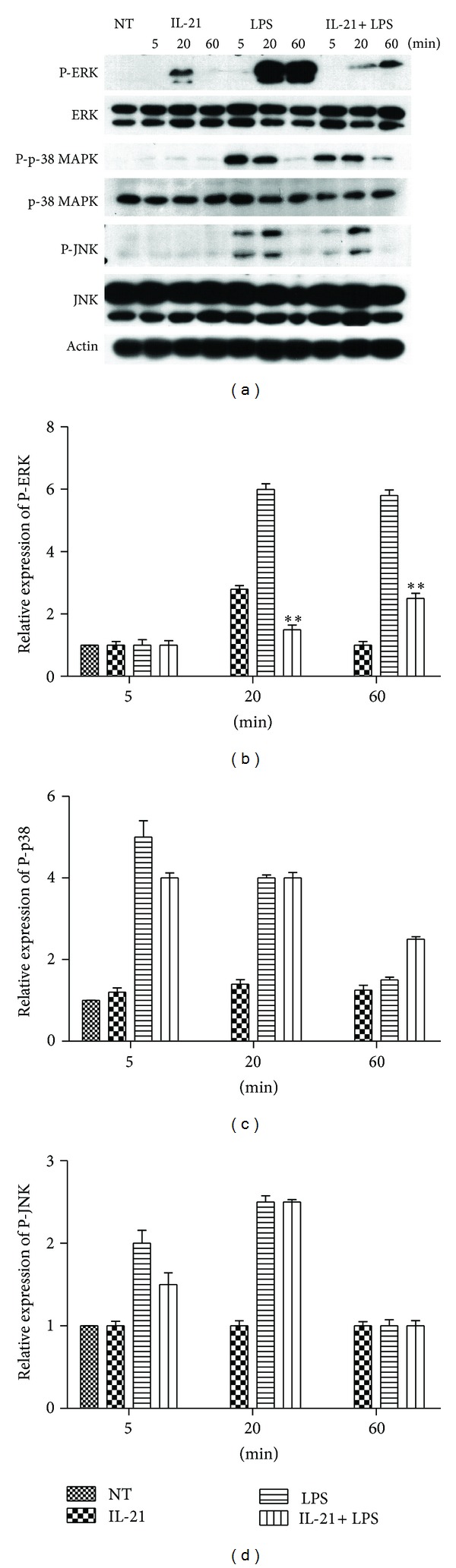
Effects of IL-21 on LPS-induced MAPKs signal pathway in mouse peritoneal macrophages. Macrophages (5 × 10^6^) were incubated with IL-21 (100 ng/mL) in the presence or absence of LPS (100 ng/mL) for 5 min, 20 min, and 60 min. Cytosolic proteins were analyzed by Western blotting for ERK1/2, p38 MAPK, and JNK phosphorylated. Detection of Actin was estimated as protein-loading control for each lane. Values are presented as mean ± SD of three independent experiments. *P < 0.05, **P < 0.01 compared with the LPS group.

The MAPK pathway plays a critical role in the regulation of cell growth and differentiation and controls cellular responses to cytokines and stresses. The expression of proinflammatory mediators (such as TNF-*α*, IL-1*β*, and IL-6) has been shown to be modulated by the MAPK pathway in LPS-induced macrophages. To examine whether the inhibition of the inflammatory response by IL-21 is also mediated through the MAPK signal pathway, the cytoplasmic protein was extracted and the phosphorylation of p38 MAPK, ERK1/2, and JNK was examined by Western blot. The results showed that IL-21 triggers the activation of ERK1/2 in 20 min, and LPS stimulation significantly increased the phosphorylation of ERK1/2, p38 MAPK, and JNK at 5, 20, and 60 min, respectively. However, IL-21 clearly inhibited the phosphorylation of ERK in LPS-induced macrophages at 20 min and 60 min (*P* < 0.01, Figures 4(a) and 4(b)). IL-21 weakly inhibited the phosphorylation of p38 MAPK and JNK at 5 min in LPS-induced macrophages (Figures 4(a), 4(c), and 4(d)). However, the levels of non-phosphorylated MAPK isoform did not vary remarkably between groups.

### 3.5. IL-21 Inhibited I*κ*B*α* Phosphorylation and NF-*κ*B Translocation in LPS-Induced Macrophages

**Figure 5:**
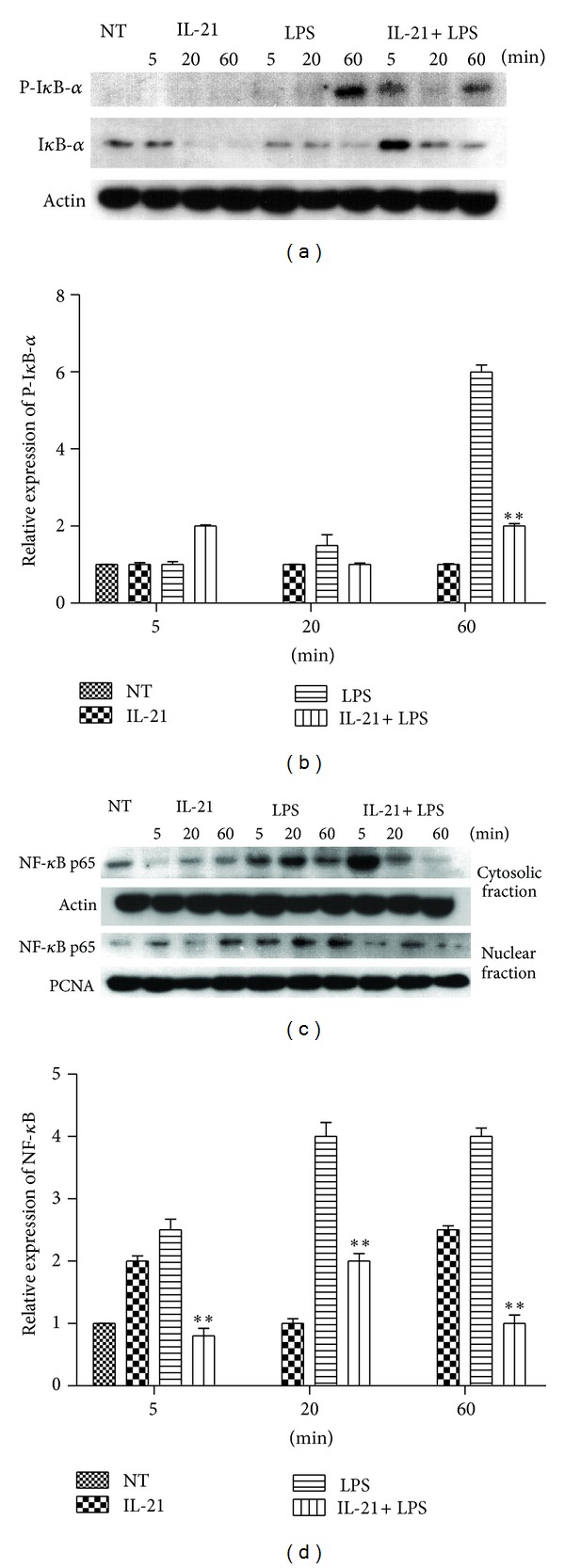
Effects of IL-21 on LPS-induced NF-*κ*B signal pathway in mouse peritoneal macrophages. Macrophages (5 × 10^6^) were incubated with IL-21 (100 ng/mL) in the presence or absence of LPS (100 ng/mL) for 5 min, 20 min, and 60 min. Nuclear and cytosolic proteins from macrophages were analyzed by Western blot with specific antibodies. Detection of Actin and PCNA was estimated as protein-loading control for each lane. Values are presented as mean ± SD of three independent experiments. *P < 0.05, **P < 0.01 compared with the LPS group.

The activation of NF-*κ*B is integral to the activation of proinflammatory mediators such as TNF-*α*, IL-6, and IL-1*β* in LPS-stimulated macrophages. The NF-*κ*B/Rel transcription factors are present in the cytosol in an inactive state complexed with the inhibitory I*κ*B proteins [16–18]. Activation occurs via the phosphorylation of I*κ*B-*α* at Ser32 and Ser36 followed by proteasome-mediated degradation, resulting in the release and nuclear translocation of active NF-*κ*B [16–18]. Therefore, the activation of NF-*κ*B was assessed in macrophages by measuring the degree of phosphorylation of I*κ*B-*α* protein. The results showed that the phosphorylation of I*κ*B-*α* was increased in LPS-induced macrophages, but it was significantly inhibited by IL-21 in 60 min (*P* < 0.01, Figures 5(a) and 5(b)). Because NF-*κ*B must be translocated to the nucleus once released by its inhibitor to activate gene transcription, the nuclear proteins were extracted and evaluated for NF-*κ*B translocation. The results showed that NF-*κ*B translocation was detected in the nuclear proteins of LPS-induced macrophages starting at 5 min and was detectable until 60 min. However, IL-21 significantly inhibited LPS-induced NF-*κ*B translocation (*P* < 0.01, Figures 5(c) and 5(d)).

### 3.6. IL-21 Prevented LPS-Induced Apoptosis in Macrophages

**Figure 6:**
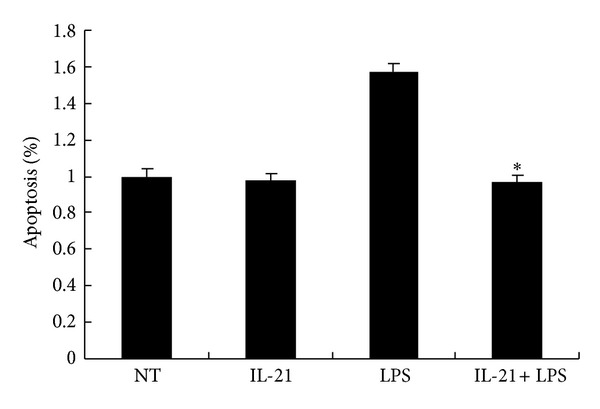
Effects of IL-21 on LPS-induced apoptosis in mouse peritoneal macrophages. Macrophages (1 × 10^6^) were incubated with IL-21 (100 ng/mL) in the presence or absence of LPS (100 ng/mL) for 24 h and were stained with FITC-conjugated Annexin V and propidium iodide (PI). Values are presented as mean ± SD of three independent experiments. *P < 0.05 compared with the LPS group.

IL-21 (1 *μ*g/mL) is reported to induce apoptosis in B lymphoma cell lines [19]. In order to evaluate whether this apoptosis is related to decreased cytokine production, macrophages (1 × 10^6^) were stained with FITC-conjugated Annexin V and propidium iodide (PI) after being cultured for 24 h with IL-21 and/or LPS. The result revealed that LPS promotes cell apoptosis compared to the NT group (*P* < 0.001). However, IL-21 clearly prevents LPS-induced apoptosis in macrophages (*P* < 0.05, Figure 6).

### 3.7. IL-21 Decreased the Population of M1 Macrophage in LPS-Induced Macrophages

**Figure 7:**
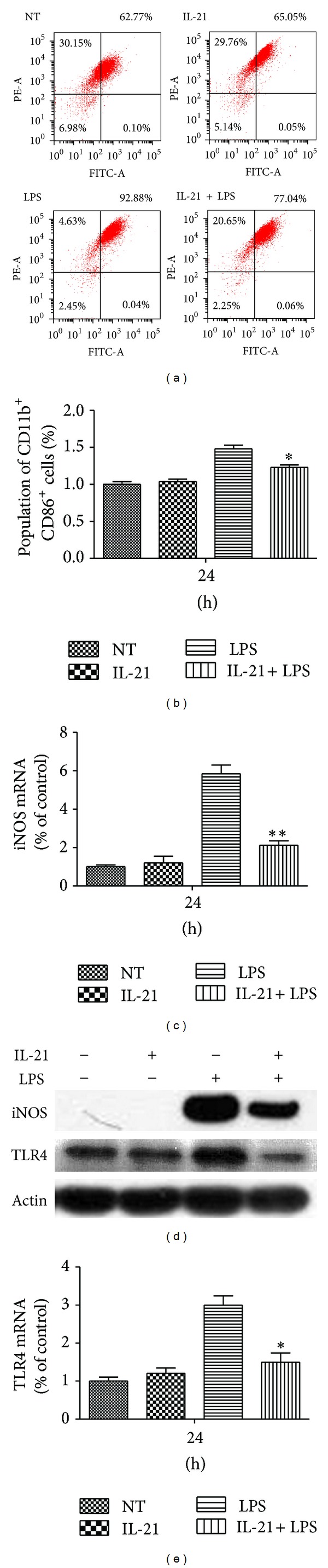
Effects of IL-21 on LPS-induced expression of cell surface markers in mouse peritoneal macrophages. Macrophages (1 × 10^6^) were incubated with IL-21 (100 ng/mL) in the presence or absence of LPS (100 ng/mL) for 24 h. (a) and (b) Gating strategy used to identify M1 macrophages present in peritoneal macrophages based on surface molecules CD11b and CD86. (c)–(e) Total proteins from macrophages were analyzed by Western blot with specific antibodies. Detection of Actin was estimated as protein-loading control for each lane. Values are presented as mean ± SD of three independent experiments. *P < 0.05, **P < 0.01 compared with the LPS group.

M1 and M2 phenotypes might not be stably differentiated subsets in the same way as, for example, Th1 and Th2 cells. The commonly held view is that macrophages can undergo dynamic transitions between different functional states driven by cues in the tissue microenvironment, which can include cytokines, growth factors, and microorganism-associated molecular patterns. In order to confirm whether IL-21 can promote a phenotype transition from M1 toward M2, the M1 macrophage surface markers CD11b and CD86 were evaluated by flow cytometry [12]. More than 92.88% of the adherent cell population was M1 macrophages as determined by specific surface marker CD11b and CD86 staining after incubation with LPS. IL-21 does not affect the population of CD11b^+^CD86^+^ cells compared to the NT group. However, IL-21 decreased the population of CD11b^+^CD86^+^ cells in LPS-stimulated cells compared to the LPS group (*P* < 0.05, Figures 7(a) and 7(b)).

The production of the M1-activation marker nitric oxide (NO) plays a critical role in many physiological processes, particularly in a host's innate immunity system to inhibit microbial growth, tumor cell growth, and tumor metastasis [20]. The release of NO is regulated by iNOS. The expression of iNOS was evaluated to confirm whether IL-21 can affect the population of M1 macrophage in LPS-induced macrophages. The result showed that the mRNA expression levels of iNOS were remarkably increased after being stimulated by LPS alone, but IL-21 treatment significantly inhibited LPS-induced mRNA and protein expression of iNOS (*P* < 0.01, Figures 7(c) and 7(d)). Moreover, IL-21 also inhibited the mRNA and protein expression of TLR4, another surface marker of M1 macrophages, in LPS-induced macrophages (*P* < 0.05, Figures 7(d) and 7(e)).

### 3.8. IL-21 Increased STAT3 Phosphorylation in LPS-Induced Macrophages

**Figure 8:**
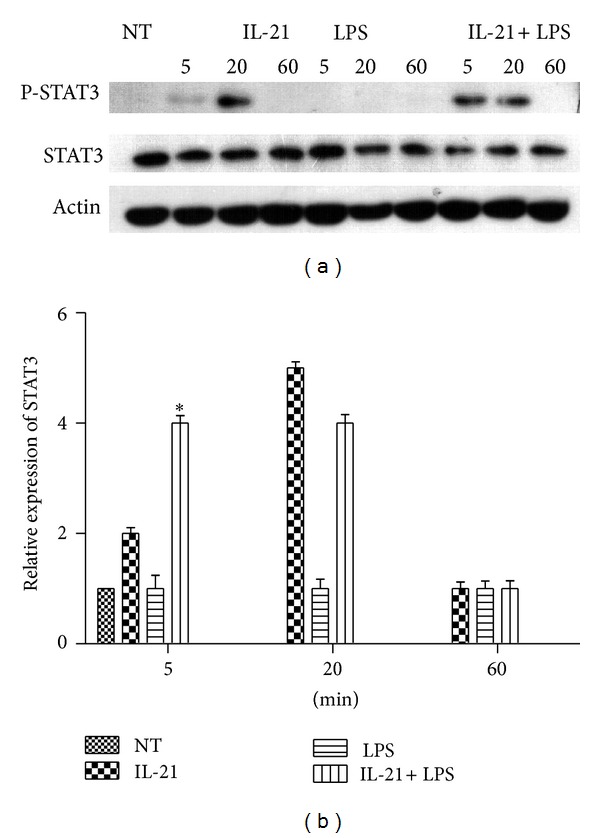
Effects of IL-21 on LPS-induced STAT3 signal pathway in mouse peritoneal macrophages. Macrophages (5 × 10^6^) were incubated with IL-21 (100 ng/mL) in the presence or absence of LPS (100 ng/mL) for 5 min, 20 min, and 60 min. Cytosolic proteins from macrophages were analyzed by Western blot with specific antibodies. Detection of Actin was estimated as protein-loading control for each lane. Values are presented as mean ± SD of three independent experiments. *P < 0.05 compared with the LPS group.

IL-21 activates both Jak1 and Jak3. However, Stat3 appears to be the most important STAT protein for IL-21 signaling. Because of a predominance of STAT3 and STAT6 activation results in M2 macrophage polarization [21], we also evaluated the STAT3 phosphorylation in LPS-induced macrophages. The results showed that treatment with IL-21 alone triggers the phosphorylation of STAT3 at 5 min and 20 min. Moreover, IL-21 clearly increases the phosphorylation of STAT3 in LPS-induced macrophages at 5 min (*P* < 0.005, Figure 8).

## 4. Discussion

IL-21 is produced by CD4^+^ T cells as well as by NK T cells and has pleiotropic effects on both innate and adaptive immune responses, such as enhancing the proliferation of lymphoid cells, increasing the cytotoxicity of CD8^+^ T cells and NK cells, and promoting the differentiation of B cells into plasma cells, the inhibitory antigen-presenting functions of DCs, and proapoptotic signals for B cells and NK cells. Biologic activities of IL-21 are mediated by a specific IL-21R, structurally related to the IL-21R chain, which is associated with the common *γ*c for signal transduction [22, 23]. The peritoneal cavity is a unique compartment within which a variety of immune cells reside and from which macrophages are commonly drawn for functional studies. In this study, we first evaluate the expression of IL-21R and *γ*c in macrophages. As shown in Figure 1, IL-21R is expressed by macrophages from mice, and its expression is upregulated by stimulation with IL-21. In addition, the expression level of *γ*c mRNA has no change after being stimulated by IL-21 and/or LPS.

The production of chemokines and cytokines such as TNF-*α*, IL-6, and IL-1*β* by activated macrophages plays a critical role in diverse physiological processes, particularly in a host's innate immunity system to inhibit microbial growth, tumor cell growth, and tumor metastasis. TNF-*α* is the earliest and primary endogenous mediator. Significant expression of TNF-*α* has been shown in many types of inflammatory processes. IL-6 is an important cytokine mediator released in many immunological and inflammatory responses [24]. IL-21 has been reported to inhibit LPS-induced cytokine production in human monocyte-derived DCs [25]. In this study, we have proven that IL-21R is expressed in peritoneal macrophages. Thus, we hypothesize that IL-21 has the potential ability to act directly on macrophages to inhibit proinflammatory cytokine and chemokine production. In order to confirm the potential anti-inflammatory effects of IL-21 *in vitro*, the production of cytokines and chemokines was studied in LPS-induced macrophages. As shown in Figure 2, the expression of cytokine and chemokine mRNA was remarkably increased in macrophages after LPS challenge, and IL-21 was able to inhibit the expression of TNF-*α*, IL-6, and IL-1*β* at 3 h, 6 h, and 24 h in LPS-induced macrophages, but not of IL-10, CCL5, IFN-*γ*, and CXCL2. Moreover, the concentrations of TNF-*α*, IL-6, and IL-1*β* in cell-free supernatant were also detected and were dramatically increased after treatment with LPS. As shown in Figure 3, IL-21 was able to suppress the production of TNF-*α* and IL-6 at 12 h and 24 h, respectively. In this paper, we detected the mRNA expression of IL-1*β*, but not protein secretion, which may be relevant to the maturation of IL-1*β*. In mammals, inflammasomes are composed of sensory NLRs, an adaptor protein called ASC, and the caspase-1 protease, and their activation results in the processing of procaspase-1 into activated caspase-1, which then cleaves pro-IL-1*β* and pro-IL-18 into the secreted cytokines IL-1*β* and IL-18 [26, 27]. The concentration of LPS used in this experiment is too low to initiate inflammasome assembly or cleave pro-IL-1*β* into the biologically active IL-1*β*. These results indicated that IL-21 plays an anti-inflammatory role in LPS-induced macrophages.

To further characterize the nature of the inhibitory effect of IL-21 on cytokine production, the MAPKs and NF-*κ*B signal transduction pathways, which were activated by LPS as well as by TNF-*α* and IL-1*β* in macrophages, were examined. First, we examined the effects of IL-21 on the phosphorylation of p38, ERK, and JNK, which have been implicated in the release of immune-related cytotoxic factors such as iNOS, COX-2, and proinflammatory cytokines in activated macrophages [28, 29]. As shown in Figure 4, LPS induced the activation of all three families of MAPKs in macrophages at different time points. However, treatment with IL-21 clearly inhibited the phosphorylation of ERK in LPS-induced macrophages. The results showed that IL-21 can inhibit cytokine production in LPS-induced macrophages, at least in part, through suppressing the phosphorylation of ERK.

In inactivated cells, NF-*κ*B is localized in the cytosol due to its binding with I*κ*B. However, when cells are activated by LPS, I*κ*B is phosphorylated by I*κ*B kinase and degraded. Then the released NF-*κ*B is translocated into the nucleus [30] where it binds to specific sequences of DNA termed response elements (RE) and induces the expression of proinflammatory factors [16–18]. Inducible genes that are known to be transactivated by NF-*κ*B include, but are not limited to, TNF-*α*, IL-1*β*, IL-6, IL-8, IFN-*γ*, iNOS, CXCL2, and CCL5 in macrophages [31]. As shown in Figure 5, the results showed that the phosphorylation of I*κ*B*α* in macrophages was increased after induction by LPS, but it was significantly inhibited by IL-21 at 60 min. In addition, NF-*κ*B translocation was detected in the nucleus of LPS-induced macrophages started at 5 min and was detectable until 60 min, but it was inhibited by IL-21 at 20 min and 60 min. These results showed that IL-21 can also inhibit cytokine production in LPS-induced macrophages, at least in part, through suppressing I*κ*B*α* phosphorylation and NF-*κ*B translocation.

IL-21 (1 *μ*g/mL) is reported to induce apoptosis in B lymphoma cell lines [19]. Because of the possibility that IL-21 may promote apoptosis in LPS-induced macrophages, an apoptosis assay was performed to confirm whether this apoptosis is relevant to the decreased cytokine production. As shown in Figure 6, IL-21 clearly prevents LPS-induced apoptosis in macrophages compared to the LPS group. The result indicated that the inhibition of cytokine production was not induced by cell apoptosis after treatment with IL-21 in LPS-induced macrophages.

Macrophage polarization is driven by cues in the tissue microenvironment, which can include cytokines, growth factors, and microorganism-associated molecular patterns. At least* in vitro*, LPS-activated macrophages after a few hours become unable to reactivate a large fraction of proinflammatory genes following restimulation [32]. However, they retain the ability to induce the expression of many other genes, including Arg1, Il10, and Mrc1 [12], for example. This altered state of responsiveness to secondary stimulation is commonly referred to as endotoxin tolerance and results in a global and sustained switch of the gene expression program from a proinflammatory M1 signature to an M2-like anti-inflammatory phenotype [33]. Thus, we evaluated the phenotype transition from M1 to M2 to confirm whether it is related to the decreased production of IL-6 and TNF-*α*. Macrophages are often distinguished from DCs by the differential expression of surface makers such as F4/80, CD11b and CD18 (also known as MAC1), CD68, and Fc receptors [34]. In addition, TLR4, CD86, and iNOS are important surface markers of M1 macrophages [35]. In this paper, cells stained with CD11b and CD86 antibodies were regarded as M1 macrophages. As shown in Figure 7, IL-21 reduced M1 populations in LPS-induced macrophages compared to the LPS group. Furthermore, IL-21 inhibited the gene and protein expression of TLR4 and iNOS in LPS-stimulated macrophages. A predominance of STAT3 and STAT6 activation results in M2 macrophage polarization, associated with immune suppression and tumor progression [21]. IL-21 activates both Jak1 and Jak3. Moreover, STAT3 appears to be the most important STAT protein for IL-21 signaling. Because of the possibility that IL-21 may promote M2 macrophages polarization, we evaluated the phosphorylation of STAT3 in LPS-induced macrophages. As shown in Figure 8, IL-21 clearly increased the activation of STAT3 in LPS-induced macrophages at 5 min and 20 min. Our results indicated that IL-21 promotes a shift from the M1 to M2 macrophage phenotype by decreasing the expression of iNOS, CD86, and TLR4 and by increasing STAT3 phosphorylation in LPS-stimulated cells.

In summary, our studies demonstrate that IL-21 promotes a shift from the M1 to M2 macrophage phenotype by decreasing the expression of iNOS, CD86, and TLR4 and by increasing STAT3 phosphorylation in LPS-stimulated cells, and leads to the decrease of the phosphorylation of ERK and translocation of NF-*κ*B and thereby reducing TNF-*α* and IL-6 production.

## Supplementary Material

Supplement 1: U0126 and BAY11-7082 inhibits IL-21-induced up-regulation of cytokine production in mouse peritoneal macrophages. Macrophages (1 × 106) were pretreated with U0126 (ERK-1/2 inhibitor) and/or BAY11-7082 (NF-*κ*B inhibitor) for 60 min prior to stimulation with IL-21 (100 ng/ml) and/or LPS (100 ng/ml) for 12 h and 24 h. Proteins expression of IL-6 and TNF-*α* were evaluated by ELISA, respectively. Values are presented as mean ± SD of three independent experiments. ^#^ *P* < 0.001 signifcantly different from the NT group; *P < 0.05, ^∗ ∗^ *P* < 0.01 compared with the LPS group.
Supplement 2: Effects of various concentration IL-21 on LPS-induced IL-6 secretion in mouse peritoneal macrophage culture supernatants Macrophages were incubated with IL-21 (20–500 ng/ml) in the presence or absence of LPS (100 ng/ml) for 12 h and 24 h. The concentration of IL-6 was measured by ELISA. Values are presented as mean ± SD of three independent experiments.
