# Supplementary material for: IL-21 Modulates Release of Proinflammatory Cytokines in LPS-Stimulated Macrophages through Distinct Signaling Pathways
Source: Mediators Inflamm. 2013 Dec 26;2013:548073. doi: 10.1155/2013/548073 (PMC3888770; doi:10.1155/2013/548073)
Supplement: Supplementary file 1 — Supplement 1: U0126 and BAY11-7082 inhibits IL-21-induced up-regulation of cytokine production in mouse peritoneal macrophages. Macrophages (1 × 10^6^) were pretreated with U0126 (ERK-1/2 inhibitor) and/or BAY11-7082 (NF-κB inhibitor) for 60 min prior to stimulation with IL-21 (100 ng/ml) and/or LPS (100 ng/ml) for 12 h and 24 h. Proteins expression of IL-6 and TNF-α were evaluated by ELISA, respectively. Values are presented as mean ± SD of three independent experiments. # P < 0.001 signifcantly different from the NT group; *P < 0.05, ∗ ∗ P < 0.01 compared with the LPS group. Supplement 2: Effects of various concentration IL-21 on LPS-induced IL-6 secretion in mouse peritoneal macrophage culture supernatants Macrophages were incubated with IL-21 (20–500 ng/ml) in the presence or absence of LPS (100 ng/ml) for 12 h and 24 h. The concentration of IL-6 was measured by ELISA. Values are presented as mean ± SD of three independent experiments. [file 548073.f1.doc]

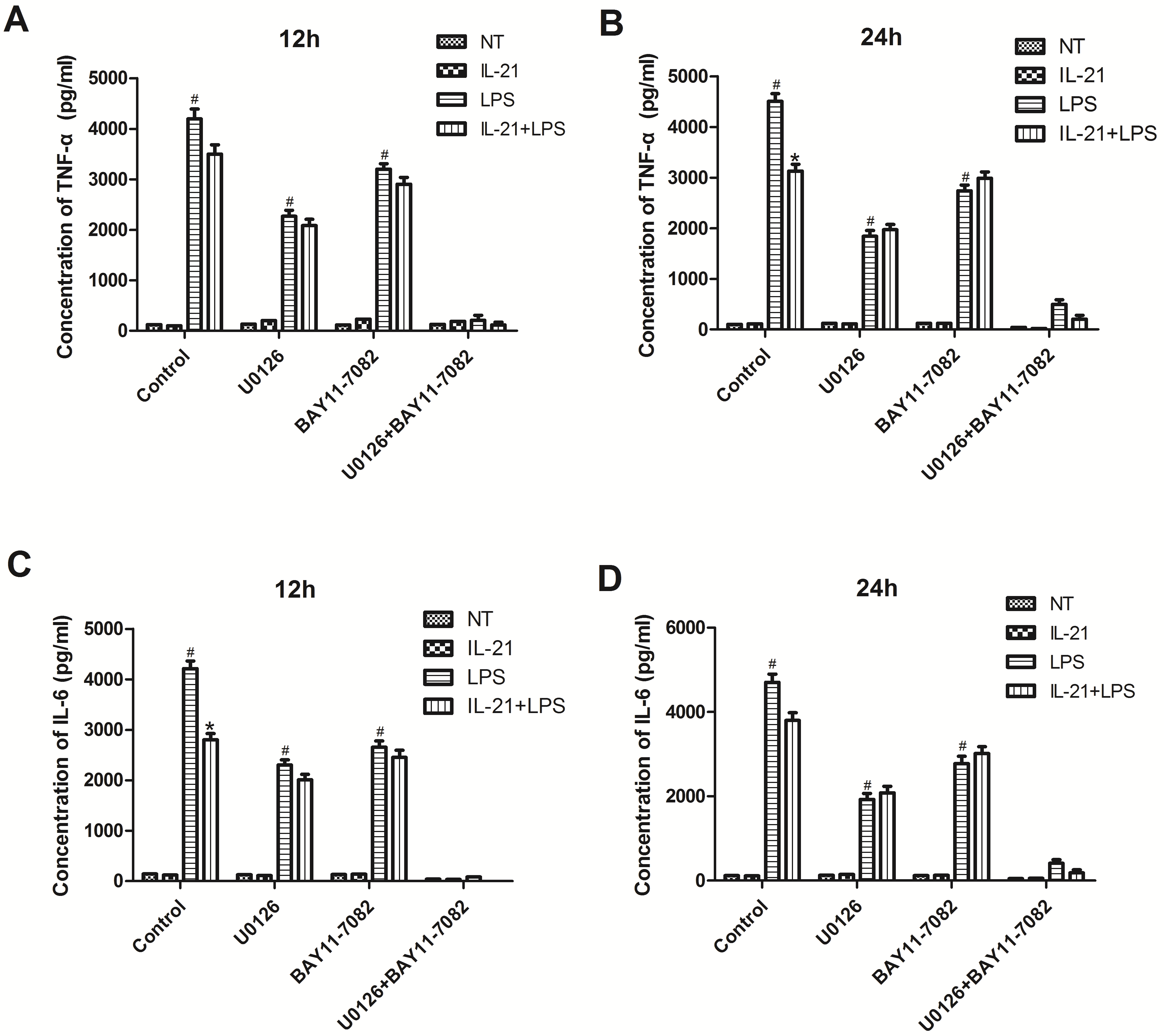


Supplement 1: U0126 and BAY11-7082 inhibits IL-21-induced up-regulation of cytokine production in mouse peritoneal macrophages. Macrophages (1×10^6^) were pretreated with U0126 (ERK-1/2 inhibitor) and/or BAY11-7082 (NF-κB inhibitor) for 60 min prior to stimulation with IL-21 (100 ng/ml) and/or LPS (100 ng/ml) for 12 h and 24 h. Proteins expression of IL-6 and TNF-α were evaluated by ELISA, respectively. Values are presented as mean ± SD of three independent experiments. # P<0.001 signiﬁcantly different from the NT group; *P<0.05, **P<0.01 compared with the LPS group.


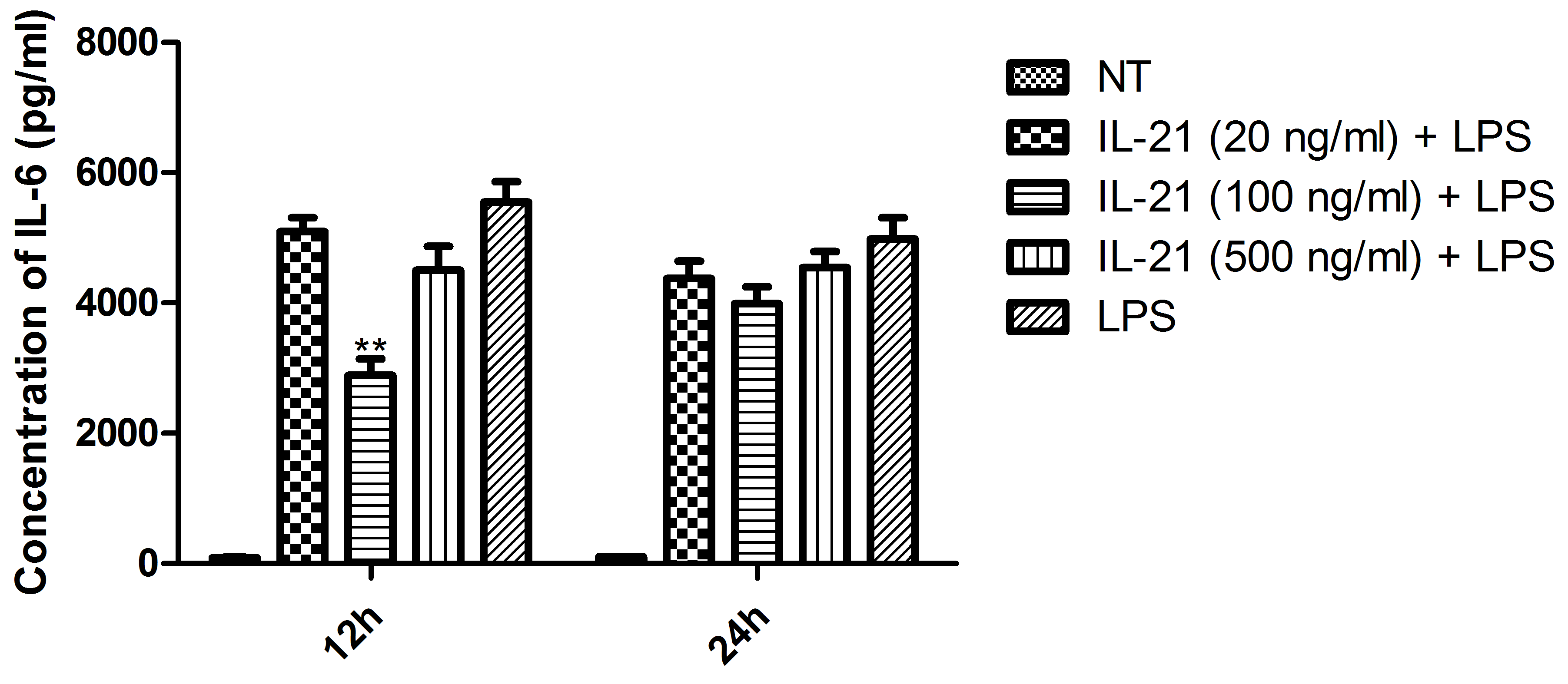


Supplement 2. Effects of various concentration IL-21 on LPS-induced IL-6 secretion in mouse peritoneal macrophage culture supernatants Macrophages were incubated with IL-21 (20-500 ng/ml) in the presence or absence of LPS (100 ng/ml) for 12 h and 24 h. The concentration of IL-6 was measured by ELISA. Values are presented as mean ± SD of three independent experiments.
